# Glycyrrhizic acid exhibits strong anticancer activity in colorectal cancer cells via SIRT3 inhibition

**DOI:** 10.1080/21655979.2021.2001925

**Published:** 2022-01-26

**Authors:** Zhenkui Zuo, Lulu He, Xiaoyu Duan, Zining Peng, Jiarui Han

**Affiliations:** a Department of Proctology, Henan Provincial Hospital of Traditional Chinese Medicine, The Second Hospital Affiliated to Henan University of Chinese Medicine, Zhengzhou, Henan Province, People’s Republic of China; b Department of Nephropathy, Henan Provincial Hospital of Traditional Chinese Medicine, The Second Hospital Affiliated to Henan University of Chinese Medicine

**Keywords:** Glycyrrhizic acid, colorectal cancer, apoptosis, anticancer, SIRT3

## Abstract

Sirtuin-3 (SIRT3) has been described as a colorectal cancer oncogene and to be regulated by glycyrrhizic acid (GA). However, few studies have explored the interaction between GA and SIRT3. Therefore, in the present study, we showed that GA could significantly decrease SIRT3 protein levels in SW620 and HT29 cells in a dose-dependent manner. Then, we overexpressed SIRT3 by lentivirus infection on SW620 and HT29 cells. We found that, *in vitro*, GA treatment significantly decreased cell viability, cell clone number, and invasion and migration number, besides significantly increasing apoptosis. Also, GA treatment significantly decreased the Bax/Bcl2 protein ratio and the expression of Cyclin D1, CDK2, CDK4, MMP-9, N-cadherin, and vimentin in SW620 and HT29 cells. Meanwhile, the SIRT3 overexpression could significantly reverse these changes. Moreover, the GA treatment could significantly decrease the weight of xenograft tumor tissues and its SIRT3 protein levels *in vivo*, while SIRT3 overexpression reversed these effects. Overall, GA inhibited the proliferation, invasion, and migration of colorectal cancer cells, and induced their apoptosis by SIRT3 inhibition.

## Introduction

Colorectal cancer is one of the most common malignant tumors, corresponding to about 1.8 million new cases and more than 800,000 deaths worldwide each year [1]. In China, 376,000 new cases and 191,000 deaths are reported each year [2,3]. Besides, due to the lack of colonoscopy in China, many colorectal cancer patients are diagnosed at an advanced stage [4,5]. Therefore, surgical resection alone is not suitable for these patients and must be combined with drug treatments.

Novel antitumor agents derived and identified from natural sources have attracted increasing attention. These compounds represent a key alternative for the improvement of existing standard cancer therapies. Glycyrrhizic acid (GA) is the main effective ingredient of licorice, a traditional Chinese medicine, which has been proven to have antioxidative stress [6], anti-inflammatory [7], anti-cancer [8], and antibacterial [9] activities. Moreover, previous studies have found that GA has a significant inhibitory effect on leukemia [10], liver cancer [11], lung cancer [12], and breast cancer cells [13]. Importantly, Khan R et al. found that oral GA could reduce the expression of Ki-67, NF-κB, proliferating cell nuclear antigen (PCNA), cyclooxygenase-2 (COX-2), and iNOS; and increase the levels of p53, connexin-43, Bcl-2, and caspase-3 proteins, thereby preventing the occurrence of 1,2-dimethylhydrazine-induced colon cancer [14,15]. Additionally, glycyrrhetnic acid, derived from licorice and with a similar structure to GA, has been found to inhibit the proliferation, invasion, and migration of colorectal cancer cells *in vitro* [16]. However, the effect of GA on colorectal cancer cells and its molecular mechanisms remain unclear.

Sirtuin-3 (SIRT3) is a NAD-dependent class III histone deacetylase. The main members of this family, such as SIRT1 [17], SIRT4 [18], and SIRT6 [19], are involved in the occurrence and development of different malignant tumors. SIRT3 has also been found to be involved in the regulation of cancer occurrence and development, However, its function can vary depending on the tumor type, being an oncogene [20] or tumor suppressor gene [21], for example. In colorectal cancer, SIRT3 is an oncogene, highly expressed in colorectal cancer tissues [20], and related to the sensitivity of colorectal cancer cells to chemotherapy drugs [22]. In the present study, we hypothesized that GA exerted its anti-colorectal cancer activity by SIRT3 inhibition. We designed this study to investigate the GA’s effects on the proliferation, invasion, migration, and apoptosis of colorectal cancer cells, and to explore the relationship between GA’s molecular mechanisms and SIRT3.

## Materials and methods

### Cell lines culture

**Figure 1.**
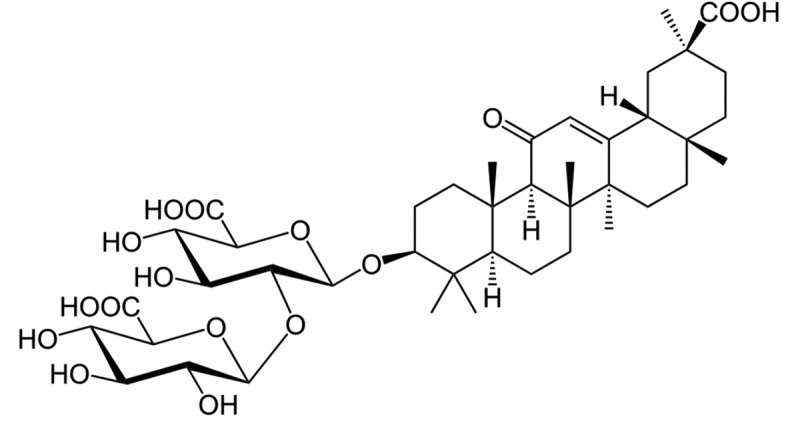
Molecular formula of glycyrrhizic acid.

SW620 (CCL-227) and HT29 (HTB-38) cell lines were purchased from the American Type Culture Collection (ATCC). They were cultured in McCoy’s 5A medium (12330031, Gbico) supplemented with 10% fetal bovine serum (16140071, Gbico) at 37°C and 5% CO2. Different concentrations of GA (1295888, Sigma) were directly added to the medium to perform treatments (GA, Figure 1).

### Western blot analyses

First, we harvested and lysed cells to obtain the total protein using the RIPA lysis buffer (R0013D, Beyotime). Then, we used a BCA kit to detect the total protein concentration and protein levels as previously described [23]. Fifty μg of total protein was analyzed with 10% SDS-PAGE. After transferring samples to PVDF membranes and sealing with 5% skimmed milk, they were incubated with the primary antibodies overnight at 4°C. After incubation with the secondary antibody (room temperature and 1 h), proteins were visualized with ECL solution (WBKLS0100, Beijing Xinjingke Biotechnologies Co., Ltd, China), followed by densitometry analyses using Image J 3.0 (IBM, USA). β-actin was also loaded as a control. The antibodies used in this study are shown in Table 1.

**Table 1.** Antibody information

Antibody	Cat.no.	Manufacturer
SIRT3	ab217319	ABCAM
Cyclin D1	ab16663	ABCAM
CDK2	ab64669	ABCAM
CDK4	GTX55565	Gene Tex
MMP-9	sc-21733	Santa Cruz
N-cadherin	sc-8424	Santa Cruz
Vimentin	ab230171	ABCAM
Bax	AF820	R&D
Bcl2	AF810	R&D
β-actin	HC201-02	TransGen Biotech
Goat anti-Rabbit IgG (H + L)-Alexa Fluor 488	A11008	Invitrogen
Goat Anti-Rabbit IgG H&L (HRP)	ab97051	ABCAM
Goat Anti-mouse IgG H&L (HRP)	Ab205719	ABCAM

### Cellular immunofluorescence

Cells (1x10^4^) were seeded into a Lab-Tek cell culture well (155411, Thermo Scientific). After 24 h of 20 μmol/L GA treatment, we removed the cell culture medium and washed cells 3 times with PBS. After being fixed with 4% paraformaldehyde for 10 min at room temperature, cells were blocked with 5% BSA for 1 h at room temperature. Then, cells were incubated with SIRT3-antibody (ab217319, abcam) overnight at 4°C. On the next day, we removed the SIRT3-antibody, and cells were incubated with Alexa Fluor488 goat-anti-rabbit IgG (H + L) (A11008, Invitrogen) after being washed 3 times with PBS. Nuclei were counterstained with 5 μg/mL DAPI for 5 min at room temperature. Finally, all samples were analyzed by confocal microscopy.

### SIRT3 overexpression

We overexpressed SIRT3 in colorectal cancer cells by AAV-h-SIRT3 (AAV-223017, vectorbiolabs) lentivirus infection. The empty vector control lentivirus (AAV-NC) was used as control. After 48 h of infection with lentivirus, we harvested cells to detect SIRT3 protein levels by Western blot.

### Cell viability assay

After 24 h of stimulation with 20 μmol/L GA, we seeded cells into a 96-well cell culture plate (1 x 10^4^ cells/well). After removing the medium, we washed cells 3 times with PBS. Then, 100 μL of cell culture medium supplemented with 10 uL CCK8 solution (C0038, Beyotime) were added to incubate cells for 1 h at 37°C and 5% CO2. Finally, we detected the OD600 to calculate the relative cell viability.

### Cell clone test

A cell clone test was used to evaluate the proliferation of colorectal cancer cells as previously described [24]. First, 200 cells with 2 mL medium were seeded into a 6-well cell culture plate. The cell culture medium was supplemented with 0 or 20 μmol/L GA. We changed the cell culture medium once every 3 days. After 3 weeks, we removed the cell culture medium and washed them twice with PBS. Then, we added formaldehyde to fix cells at room temperature for 30 min, then added 0.1% crystal violet for staining at room temperature for 20 min, and washed cells 3 times with PBS. Finally, we counted the cell clone number.

### Flow cytometry detection

After 24 h of 20 μmol/L GA stimulation, cells were harvested. For cell cycle detection, we washed cells twice with pre-cooled PBS, added pre-cooled 75% ethanol, and fixed them at 4°C for 4 h. Then we harvested cells to perform the PI/RNase staining according to the Cell Cycle Staining Kit instructions (KTA2020, Amyjet Scientific). For apoptosis detection, after being washed twice with cold-PBS, we harvested cells to perform the Annexin V-FITC/PI staining following the Annexin V-FITC/PI apoptosis detection kit instructions (40302ES20, YEASEN).

### Transwell chamber for invasion and migration assessment

We used a transwell chamber test to determine the invasion and migration of colorectal cancer cells [24]. We seeded 0.5 × 10^5^ cells/100 μL medium into the transwell upper chamber. The lower chamber received 600 uL of McCoy’s 5A medium containing 2.5% FBS. After incubation for 24 h at 37°C and 5% CO2, we removed the cell culture medium in the transwell lower chamber, washed it twice with sterile PBS, and fixed it with formaldehyde at room temperature for 30 min. Then, we added 0.1% crystal violet for staining at room temperature for 20 min and washed it 3 times with PBS. Finally, we counted cells under a microscope.

### Caspase 3 activity assay

After stimulation with 20 μmol/L GA for 24 h, we harvested cells, then detected the caspase 3 activity using a Caspase 3 Activity Assay Kit (C1116, Beyotine) and following the manufacturer’s instructions.

### Nude mouse xenograft tumor

Forty-eight nude mice (6–8 weeks, female:male = 1:1, 16–25 g) were used to establish colorectal cancer xenograft tumor models. First, we injected 2 × 10^5^ wild type (WT) or SIRT3 colorectal cancer cells cell subcutaneously under the nude mouse armpit. Nude mice in GA and GA+AAV-SIRT3 groups received 50 mg/kg of GA by gavage every day. Mice in the WT group received the same amount of solvent every day. All mice eat freely for 3 weeks, then were euthanized to separate and weigh colorectal cancer xenograft tumor tissues.

### SIRT3 expression by immunochemistry

First, colorectal cancer xenograft tumor tissues were cut into 3.5 μm slices using Slicer (CUT4062, SLEE). Then, we detected SIRT3 protein levels using an immunochemistry VECTASTAIN® Elite® ABC Kit (PK-000, Lumiprobe) following the manufacturer’s instructions.

### Statistical analyses

We used GraphPad Prism 5 to analyze the data and construct images. We used the Student’s t-test to compare differences between two groups. Differences between multiple groups were analyzed by one-way ANOVA with Tukey’s post hoc test. A *p* < 0.05 was considered as a significant difference.

## Results

In the current study, we hypothesized that GA exerted its anti-colorectal cancer activity by SIRT3 inhibition. First, we tested the effect of GA on SIRT3 protein expression in SW620 and HT29 cells. We found that GA inhibited SIRT3 expression in colorectal cells in a dose-dependent manner. Next, we found that GA inhibited the proliferation, cell cycle progression, migration, and invasion of colorectal cancer cells *in vitro*. Moreover, overexpression of SIRT3 could attenuate GA’s effects. Finally, we found that GA reduced the weight of colorectal cancer xenograft tumor tissues.

### GA inhibits SIRT3 protein expression in colorectal cancer cells

**Figure 2.**
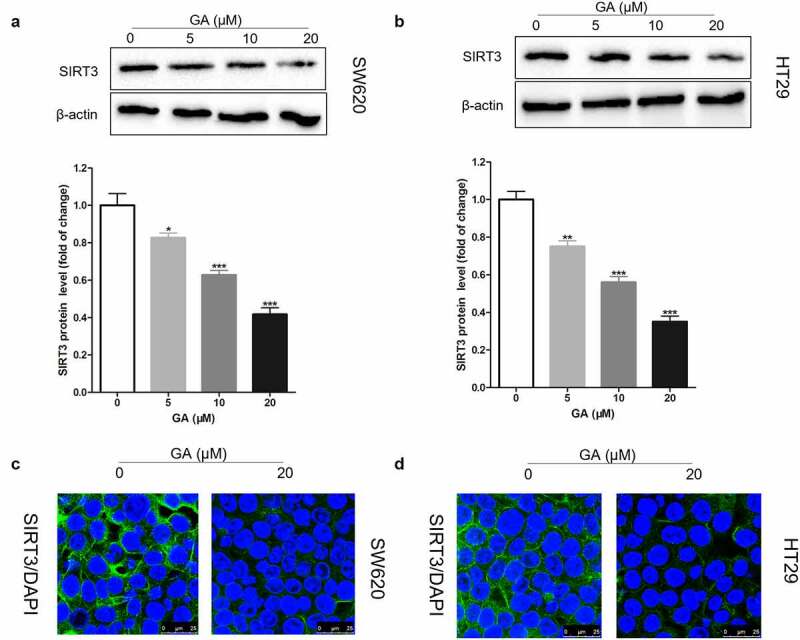
Glycyrrhizic acid inhibits SIRT3 protein expression in colorectal cancer cells. (a,b) Western blot analysis of SIRT3 protein expression in SW620 (a) and HT29 (b) cells after stimulating with 0, 5, 10 or 20 μmol/L glycyrrhizic acid for 24 hours. (c,d) Immunofluorescence analysis shows that SIRT3 is mainly expressed in the cytoplasm of SW620 (c) and HT29 (d) cells, and decreased expression of SIRT3 protein after stimulating with 20 μmol/L glycyrrhizic acid for 24 hours. Each test is repeated at least 3 times independently. * P < 0.05, ** P < 0.01 and *** P < 0.001 vs 0 μmol/L glycyrrhizic acid group.

It has been found that SIRT3 is highly expressed in colorectal cancer tissues and that SIRT3 gene silencing can inhibit the proliferation, invasion, and migration of colorectal cancer cells, besides increasing their apoptosis [20]. Here, we exposed colorectal cancer cells to 0, 5, 10, or 20 μmol/L GA for 24 h and collected them to analyze SIRT3 protein levels by Western blot. We found that GA incubation significantly reduced SIRT3 protein levels in SW620 (Figure 2(a)) and HT29 (Figure 2(b)) cell lines in a dose-dependent manner. Also, we located where SIRT3 was expressed in colorectal cancer cells using immunofluorescence. We found that SIRT3 was expressed in the cytoplasm and 20 μmol/L GA significantly decreased SIRT3 expression in SW620 (Figure 2(c)) and HT29 (Figure 2(d)) cells.

### GA inhibits the proliferation of colorectal cancer cells by SIRT3 inhibition

**Figure 3.**
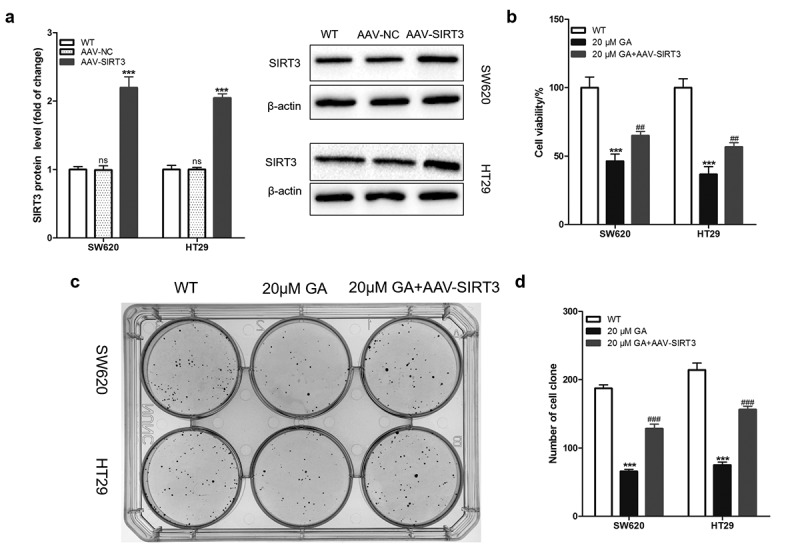
Glycyrrhizic acid inhibits the proliferation of colorectal cancer cells by inhibiting SIRT3 in vitro. (a) Lentiviral infection increases the expression of SIRT3 protein in colorectal cancer cells using Western blot analysis. (b) SIRT3 overexpression increases decreased the cell viability of colorectal cancer induced by 20 μmol/L glycyrrhizic acid for 24 hours. (c,d) Wild-type and SIRT3 overexpressing colorectal cancer cells were cultured with 0 or 20 μmol/L glycyrrhizic acid for 2 weeks, representative cell clone images (c), and statistical comparison of the number of cell clones (d). Each test is repeated at least 3 times independently. ns P > 0.05 and *** P < 0.001 vs WT group, and ### P < 0.001 vs 20 μM GA group.

To study whether GA can affect the biological characteristics of colorectal cancer cells by inhibiting SIRT3 expression, we first overexpressed SIRT3 in colorectal cancer cells through lentivirus infection (AAV-SIRT3). The Western blot results showed that the AAV-SIRT3 lentivirus infection successfully established SIRT3-overexpressed SW620 and HT29 cell lines (Figure 3(a)). Then, we exposed WT and SIRT3-overexpressed colorectal cancer cells to 0 or 20 μmol/L GA for 24 h and used a CCK8 assay kit to evaluate the cell viability. Results showed that, compared to the WT group (colorectal cancer cells without any treatment), 20 μmol/L GA significantly decreased the viability of SW620 and HT29 colorectal cancer cells (Figure 3(b)). Moreover, the overexpression of SIRT3 significantly attenuated this decrease in viability (Figure 3(b)). Furthermore, the cell clone formation test was used to assess the proliferation of colorectal cancer cells (Figure 3(c)). Results showed that 20 μmol/L GA significantly decreased the number of SW620 and HT29 cell clones (Figure 3(d)). Also, overexpression of SIRT3 significantly reduced the decrease in cell clones number induced by 24 h of 20 μmol/L GA (Figure 3(d)).

### GA hinders the progression of the colorectal cancer cell cycle via SIRT3 inhibition

**Figure 4.**
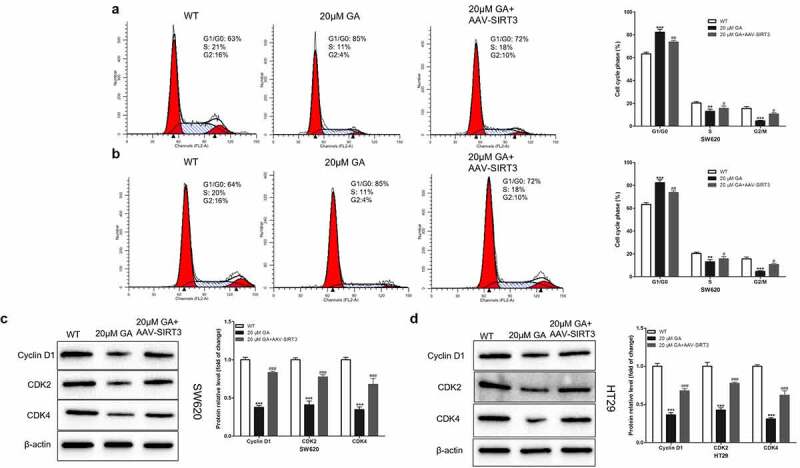
Glycyrrhizic acid blocks colorectal cancer cells in G1/G0 stage by inhibiting SIRT3 in vitro. (a,b) Flow cytometry analysis of cell cycle in SW620 (a) and HT29 (b) cells, Representative cell cycle diagrams are shown on the left, and statistical comparison cubes are shown on the right. (c,d) Western blot analysis of Cyclin D1, CDK2 and CDK4 protein expression in SW620 (c) and HT29 (d) cells after stimulating with 0 or 20 μmol/L glycyrrhizic acid for 24 hours. Each test is repeated at least 3 times independently. ** P < 0.01 and *** P < 0.001 vs WT group. # P < 0.05, ## P < 0.01 and ### P < 0.001 vs 20 μM GA group.

Since cell proliferation is closely related to the cell cycle, we hypothesized that GA affected colorectal cancer cell proliferation by blocking the cell cycle progression. To test this hypothesis, we used flow cytometry to detect the cell cycle of WT and SIRT3-overexpressed (AAV-SIRT3) colorectal cancer cells after stimulation with 0 or 20 μmol/L GA for 24 h. We found that, compared to the WT group, 20 μmol/L GA significantly increased the G1/G0 stage ratio and decreased the S/G2 stage ratio of SW620 (Figure 4(a)) and HT29 (Figure 4(b)) colorectal cancer cells. Meanwhile, overexpression of SIRT3 significantly attenuated this G1/G0 stage ratio increase and the S/G2 stage ratio decrease (Figure 4(a,b)). Moreover, we detected the changes in the expression of cell cycle-related proteins, such as cyclin D1, CDK2, and CDK4. Results showed that the stimulation with 20 μmol/L GA for 24 h significantly decreased the expression of these proteins in colorectal cancer cells compared to the WT group (Figure 4(c,d)). On the other hand, the overexpression of SIRT3 significantly attenuated this decrease in the cyclin D1, CDK2, and CDK4 protein levels.

### GA inhibits the invasion and migration of colorectal cancer cells by inhibiting SIRT3

**Figure 5.**
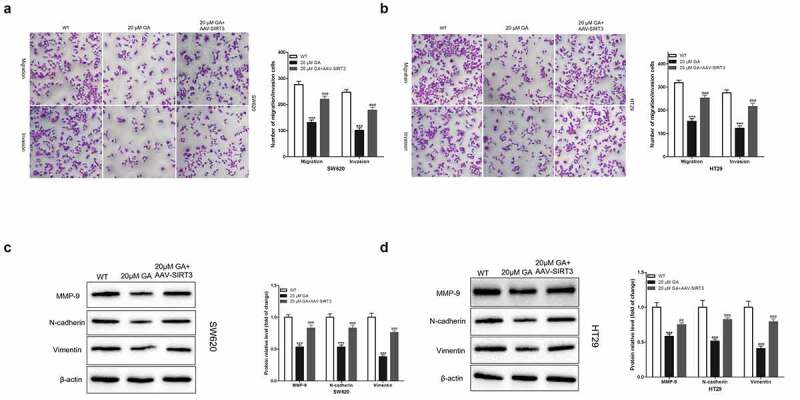
Glycyrrhizic acid inhibits the metastasis of colorectal cancer cells by inhibiting SIRT3 in vitro. (a,b) Transwell chamber was used to assess the invasion and migration ability of SW620 (a) and HT29 (b) cells. (c,d) Western blot analysis of MMP-9, N-cadherin and Vimentin protein expression in SW620 (c) and HT29 (d) cells after stimulating with 0 or 20 μmol/L glycyrrhizic acid for 24 hours. Each test is repeated at least 3 times independently. *** P < 0.001 vs WT group. ## P < 0.01 and ### P < 0.001 vs 20 μM GA group.

The invasion and migration of tumor cells endow cancer cells with malignant characteristics and are the main cause of death in tumor patients. Thus, we used a transwell chamber to evaluate the invasion and migration ability of colorectal cancer cells *in vitro*. We found that, compared to the WT group, the stimulation with 20 μmol/L GA for 24 h significantly decreased the number of SW620 (Figure 5(a)) and HT29 (Figure 5(b)) colorectal cancer cells that could invade or migrate to the lower transwell chamber. Also, overexpression of SIRT3 significantly attenuated this decrease in colorectal cancer cells number that could invade or migrate the transwell lower chamber. Also, we detected changes in the expression of epithelial-mesenchymal transition (EMT)-related proteins, such as MMP-9, N-cadherin, and Vimentin. We found that the stimulation with 20 μmol/L GA for 24 h significantly decreased the expression of these proteins in colorectal cancer cells compared to the WT group. Moreover, the overexpression of SIRT3 significantly attenuated this decrease in MMP-9, N-cadherin, and Vimentin protein levels induced by 20 μmol/L GA for 24 h (Figure 5(c,d)).

### GA induces colorectal cancer cells apoptosis by inhibiting SIRT3

**Figure 6.**
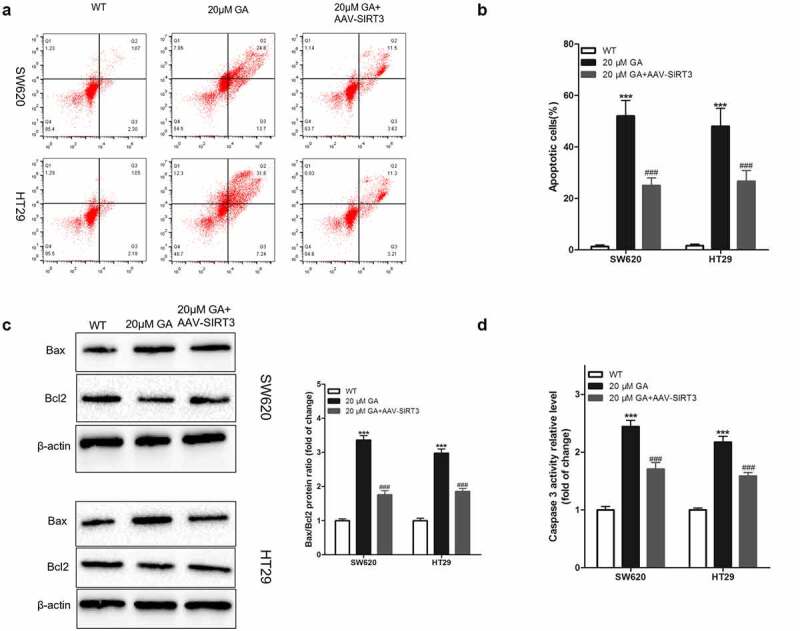
Glycyrrhizic acid induces the apoptosis of colorectal cancer cells by inhibiting SIRT3 in vitro. (a,b) 0 or 20 μmol/L glycyrrhizin stimulated colorectal cancer cells for 24 hours, flow cytometry was used to detect the apoptosis of colorectal cancer cells (a) and statistical comparison (b). (c) Western blot analysis of Bax and Bcl2 protein expression in colorectal cancer cells after stimulating with 0 or 20 μmol/L glycyrrhizic acid for 24 hours. (d) Elisa kit was used to determine the activity of caspase 3 in colorectal cancer cells after stimulating with 0 or 20 μmol/L glycyrrhizic acid for 24 hours. Each test is repeated at least 3 times independently. *** P < 0.001 vs WT group, and ### P < 0.001 vs 20 μM GA group.

The evaluation of the induction of cancer cells apoptosis is pivotal during studies with drugs used to treat cancer. Therefore, in the present study, we used flow cytometry to detect the apoptosis of colorectal cancer cells after GA treatment. We found that, compared to the WT group, the stimulation with 20 μmol/L GA for 24 h significantly increased the apoptosis of colorectal cancer cells, and overexpression of SIRT3 attenuated this effect (Figure 6(a,b)). Additionally, we detected the changes in the expression of apoptosis-related proteins, such as Bax, Bcl2, and caspase 3. Compared to the WT group, the stimulation with 20 μmol/L GA for 24 h significantly increased the Bax/Bcl2 protein ratio, and overexpression of SIRT3 attenuated this effect. Similarly, this GA treatment significantly increased the activity of caspase 3 in colorectal cancer cells compared to the WT group (Figure 6(d)). Meanwhile, the overexpression of SIRT3 significantly reversed this activity increase (Figure 6(d)).

### GA inhibits the growth of colorectal cancer xenografts via SIRT3 inhibition

**Figure 7.**
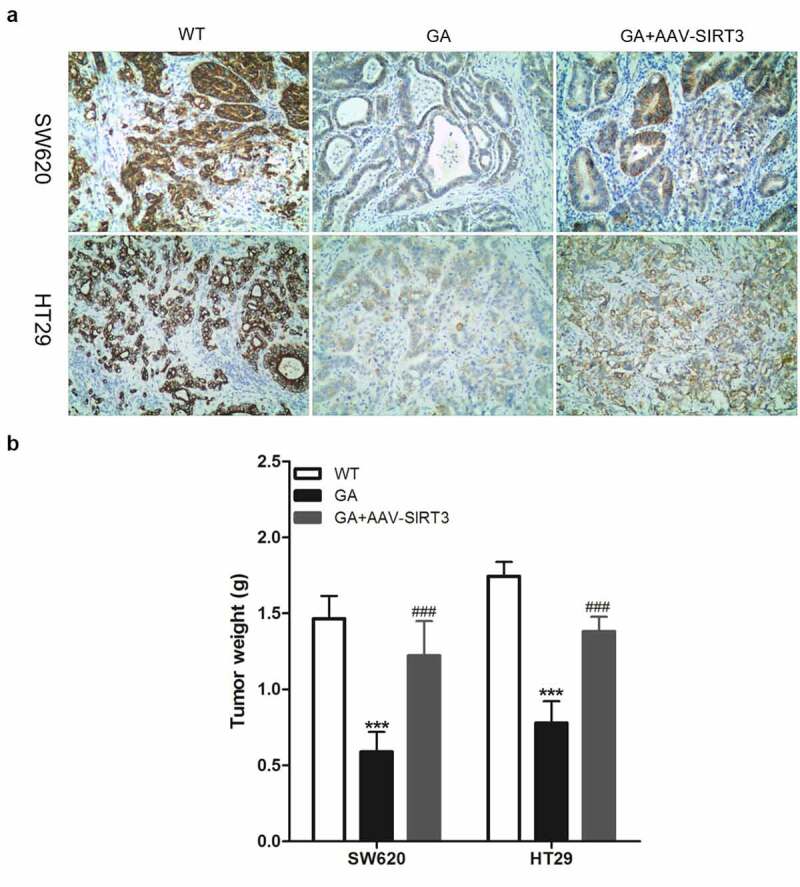
Glycyrrhizic acid inhibits SITR3 expression and the proliferation of colorectal cancer cells in nude mouse. (a-b) Subcutaneous injection of colorectal cancer cells to establish xenograft tumor models in nude mice. 3 weeks later, nude mice were euthanized, xenograft tumors were isolated, SIRT3 protein expression was detected by immunohistochemistry (a), and xenograft tumors were weighed for comparison (b). 8 mice per group. *** P < 0.001 vs WT group, and ### P < 0.001 vs 20 μM GA group.

Due to the *in vivo* environment complexity, many *in vitro* results can not be repeated *in vivo*. Thus, to verify whether GA’s effects on colorectal cancer cells are also effective *in vivo*, we established a nude mouse colorectal cancer xenograft animal model. Colorectal cancer cells were subcutaneously injected and the mice were euthanized after 3 weeks to obtain colorectal cancer xenograft tissues. Compared to the WT group, the GA treatment group presented significant decreases regarding SIRT3 protein levels in colorectal cancer xenograft tissues. However, SIRT3 protein levels in the GA+AAV-SIRT3 group were significantly higher compared to the GA group (Figure 7(a)). Moreover, the weight of colorectal cancer xenograft tissues in the GA group was significantly lower than in the WT group. Meanwhile, the weight of colorectal cancer xenograft tissues in the GA+AAV-SIRT3 group was significantly higher than the GA group (Figure 7(b)).

## Discussion

In the present study, we found that GA significantly decreased SIRT3 protein levels in colorectal cancer cells. The silencing information regulator 3 (SIRT3) is one of the seven sirtuins family members of mammals and is homologous to the yeast silence information regulator sir2. Sirtuins (SIRT1-7) are NAD-dependent deacetylated proteins and ADP ribosyltransferases and the main mediators of non-histone acetylation, Therefore, their enzymatic activity is regulated by the NAD/NADH ratio in cells. Moreover, the SIRT3 function can vary between tumor types and may even oppose [21,25]. For example, Kim HS, et al. found that SIRT3 knockout mice had a significant increase in the incidence of breast cancer in adulthood [26], and Li R, et al. found that SIRT3 has a tumor suppressor role in prostate cancer [27]. Additionally, SIRT3 can have an oncogene role in cancer. Dds T, et al found that SIRT3 was highly expressed in oral cancer and promoted the proliferation and survival of oral cancer cells [28]. In colorectal cancer, SIRT3 is highly expressed in colorectal cancer tissues and the 5-year survival rate of colorectal cancer patients with high SIRT3 expression is lower compared to those with low SIRT3 expression [20]. Additionally, Torrens-Mas M, et al. found that the SIRT3 knockdown could significantly increase oxaliplatin efficacy in SW620 cells. Therefore, SIRT3 is a colorectal cancer oncogene, and GA might exhibit its potential anticancer activity in colorectal cancer by decreasing SIRT3 expression.

To study the relationship between GA’s anti-cancer properties and SIRT3, we established SIRT3-overexpressed colorectal cancer cell lines. Then, we found that GA inhibited the proliferation of colorectal cancer cells *in vitro* and *in vivo*, while the overexpression of SIRT3 reversed this effect. We also found that GA blocks colorectal cancer cells in the G1/G0 phase, inhibiting their proliferation via SIRT3 inhibition. Since SIRT3 is a mitochondrial protein, it also affects mitochondrial energy metabolism. During proliferation, the cell needs to synthesize a lot of proteins and consume a lot of energy [29]. Therefore, SIRT3 might also control cell proliferation by regulating energy metabolism [30]. Thus, GA treatment might lead to an abnormal cell energy metabolism by SIRT3 inhibition, thereby blocking tumor proliferation. Regarding the molecular mechanisms, we found that GA significantly decreased the expression of cyclin D1, CDK2, and CDK4 proteins, and the overexpression of SIRT3 increased their expression. Cyclins [31], cyclin-dependent kinases (CDKs) [32], and cyclin-dependent kinases inhibitors (CDKIs) [33] are important in the regulation of the G1/S phase of the cell cycle. Cyclins (cyclin D1) and CDKs (CDK2 and CDK4) form a complex to promote the transition of cells from the G1 to the S phase, thereby promoting cell proliferation when cyclin D1 and CDKs are abundant [34,35]. Therefore, GA might block cell cycle progression through SIRT3-mediated cell cycle-related proteins expression.

Moreover, the migration and invasion of tumor cells to surrounding tissues characterizes the beginning and the key link of cancer metastasis, the main cause of cancer-related deaths [36]. Thus, we detected the effect of GA on the invasion and migration of colorectal cancer cells and their relationship with SIRT3 protein levels. We found that GA significantly decreased the invasion and migration of colorectal cancer cells and the overexpression of SIRT3 weakened this effect. Epithelial-mesenchymal transition (EMT) gives cells the ability to migrate and invade. In our study, we found that GA significantly decreased EMT-related proteins expression [37]. These results indicated that GA inhibited the invasion and migration of colorectal cancer cells by inhibiting SIRT3 expression.

SIRT3 is also related to apoptosis [38]. Previous studies have found that downregulation of SIRT3 promotes the apoptosis of alveolar epithelial cells [39] and EC9706 cells [40]. Besides, SIRT3 protects tumor cells against apoptosis in unfavorable environments, such as hypoxia [41] and high glucose [42]. In our current study, we found that GA induced the apoptosis of colorectal cancer cells via Bax, Bcl2, and caspase 3 inhibition, crucial proteins in the mitochondrial apoptosis pathway. Bax and caspase 3 promote apoptosis, and Bcl2 inhibits [43]. We showed that GA upregulated the expression of pro-apoptotic proteins (Bax and caspase 3) and downregulated the expression of the anti-apoptotic protein Bcl2 trough SIRT3 inhibition.

Finally, our *in vitro* results were confirmed *in vivo*, since GA significantly decreased the SIRT3 protein levels in colorectal cancer xenograft tissues along with their weights. Also, overexpression of SIRT3 reversed GA inhibitory effects on the proliferation of colorectal cancer cells *in vivo*.

## Conclusion

Overall, GA inhibited the proliferation, invasion, and migration of colorectal cancer cells, and induced their apoptosis by inhibiting SIRT3. Therefore, GA can be a potential drug for colorectal cancer treatment.

## Data Availability

The data used to support the findings of this study are available from the corresponding author upon request. The corresponding author's email is hanjiarui2018@yeah.net.The article URL is  https://www.tandfonline.com/doi/figure/10.1080/21655979.2021.2001925?scroll=top&needAccess=true

## HIGHLIGHTS

- The first discovery of GA’s anti-colon cancer activity.
- GA affects colon cancer by SIRT3 inhibition
- GA can inhibit the proliferation of colon cancer

## References

[1] Siegel RL, Miller KD, Fuchs HE, et al. Cancer statistics, 2021. CA Cancer J Clin. 2021;71:7-33. 10.3322/caac.21654 33433946

[2] Feng RM, Zong YN, Cao SM, et al. Current cancer situation in China: good or bad news from the 2018 global cancer statistics? Cancer Commun. 2019;39:1–12. 10.1186/s40880-019-0368-6 PMC6487510 31030667

[3] Chen W, Zheng R, Baade PD, et al. Cancer statistics in China, 2015. CA Cancer J Clin. 2016;66:115–132. 26808342 10.3322/caac.21338

[4] Zhang Y, Chen Z, Li J. The current status of treatment for colorectal cancer in China: a systematic review. Medicine. 2017;96:1-6. 10.1097/MD.0000000000008242 PMC5738019 28984783

[5] Huang HY, Shi JF, Guo LW, et al. Expenditure and financial burden for the diagnosis and treatment of colorectal cancer in China: a hospital‐based, multicenter, cross‐sectional survey. Cancer Commun. 2017;36:1–15. 10.1186/s40880-017-0209-4 PMC5410077 28454595

[6] Zhu L, Wei M, Yang N, et al. Glycyrrhizic acid alleviates the meconium-induced acute lung injury in neonatal rats by inhibiting oxidative stress through mediating the Keap1/Nrf2/HO-1 signal pathway. Bioengineered. 2021;12:2616–2626. 34499011 10.1080/21655979.2021.1937445 PMC8806485

[7] Zyca B, Yzl A, Jml A, et al. Glycyrrhizic acid as an adjunctive treatment for depression through anti-inflammation: a randomized placebo-controlled clinical trial. J Affect Disord. 2020;265:247–254. 32090748 10.1016/j.jad.2020.01.048

[8] Lin S-C, Chu P-Y, Liao W-T, et al. Glycyrrhizic acid induces human MDA-MB-231 breast cancer cell death and autophagy via the ROS-mitochondrial pathway. Oncol Rep. 2018;39:703–710. 29207188 10.3892/or.2017.6123

[9] Sahin F, Oznurhan F. Antibacterial efficacy and remineralization capacity of glycyrrhizic acid added casein phosphopeptide‐amorphous calcium phosphate. Microsc Res Tech. 2020;83:744–754. 32191375 10.1002/jemt.23465

[10] He SQ, Gao M, Fu YF, et al. Glycyrrhizic acid inhibits leukemia cell growth and migration via blocking AKT/mTOR/STAT3 signaling. Int J Clin Exp Pathol. 2015;8:5175. 26191214 PMC4503086

[11] Tang ZH, Li T, Chang LL, et al. Glycyrrhetinic Acid triggers a protective autophagy by activation of extracellular regulated protein kinases in hepatocellular carcinoma cells. J Agric Food Chem. 2014;62:11910–11916. 25403108 10.1021/jf503968k PMC4264863

[12] Zhu J, Chen M, Chen N, et al. Glycyrrhetinic acid induces G1-phase cell cycle arrest in human non-small cell lung cancer cells through endoplasmic reticulum stress pathway. Int J Oncol. 2015;46:981–988. 25573651 10.3892/ijo.2015.2819 PMC4324580

[13] Wang XF, Zhou QM, Lu YY, et al. Glycyrrhetinic acid potently suppresses breast cancer invasion and metastasis by impairing the p38 MAPK-AP1 signaling axis. Expert Opin Ther Targets. 2015;19:577. 25828376 10.1517/14728222.2015.1012156

[14] Khan R, Rehman MU, Khan AQ, et al. Glycyrrhizic acid suppresses 1, 2‐dimethylhydrazine‐induced colon tumorigenesis in Wistar rats: alleviation of inflammatory, proliferation, angiogenic, and apoptotic markers. Environ Toxicol. 2018;33:1272–1283. 30255981 10.1002/tox.22635

[15] Rehan K, Quaiyoom KA, Abdul L, et al. Glycyrrhizic acid suppresses the development of precancerous lesions via regulating the hyperproliferation, Inflammation, Angiogenesis and apoptosis in the colon of wistar rats. Plos One. 2013;8:e56020. 23457494 10.1371/journal.pone.0056020 PMC3573076

[16] Wang S, Yong S, Qiu R, et al. 18β-glycyrrhetinic acid exhibits potent antitumor effects against colorectal cancer via inhibition of cell proliferation and migration. Int J Oncol. 2017;51:615. 28656212 10.3892/ijo.2017.4059

[17] Han L, Long Q, Li S, et al. Senescent stromal cells promote cancer resistance through SIRT1 loss-potentiated overproduction of small extracellular vesicles. Cancer Res. 2020;80(canres.0506.2020):3383–3398. 10.1158/0008-5472.CAN-20-0506 PMC7611217 32366480

[18] Xing J, Li J, Fu L, et al. SIRT4 enhances the sensitivity of ER‐positive breast cancer to tamoxifen by inhibiting the IL‐6/STAT3 signal pathway. Cancer Med. 2019;8:7086–7097. 31573734 10.1002/cam4.2557 PMC6853819

[19] Vitiello M, Zullo A, Servillo L, et al. Multiple pathways of SIRT6 at the crossroads in the control of longevity, cancer, and cardiovascular diseases. Ageing Res Rev. 2017;35:301–311. 27829173 10.1016/j.arr.2016.10.008

[20] Liu C, Huang Z, Hong J, et al. The Sirtuin 3 expression profile is associated with pathological and clinical outcomes in colon cancer patients. Biomed Res Int. 2014;2014:871263. 25105144 10.1155/2014/871263 PMC4101237

[21] Chen Y, Fu LL, Wen X, et al. Sirtuin-3 (SIRT3), a therapeutic target with oncogenic and tumor-suppressive function in cancer. Cell Death Dis. 2014;5:e1047. 24503539 10.1038/cddis.2014.14 PMC3944233

[22] Torrens‐Mas M, Hernández‐López R, Oliver J, et al. 3 silencing improves oxaliplatin efficacy through acetylation of MnSOD in colon cancer. J Cell Physiol. 2018;233:6067–6076. 29323702 10.1002/jcp.26443

[23] Lu Q, Liu Z, He W, et al. Protective effects of ulinastatin on rats with acute lung injury induced by lipopolysaccharide. Bioengineered. 2021. DOI:10.1080/21655979.2021.1987083 PMC10813561 34637694

[24] Zhang H, Fang Z, Guo Y, et al. Long noncoding RNA SNHG10 promotes colorectal cancer cells malignant progression by targeting miR-3690. Bioengineered. 2021;12:6010–6020. 34477483 10.1080/21655979.2021.1972199 PMC8806477

[25] SIRT3. Oncogene and tumor suppressor in cancer. Cancers (Basel). 2017;9:90. 10.3390/cancers9070090 PMC5532626 28704962

[26] Kim HS, Patel K, Muldoon-Jacobs K, et al. SIRT3 is a mitochondria-localized tumor suppressor required for maintenance of mitochondrial integrity and metabolism during stress. Cancer Cell. 2010;17:41–52. 20129246 10.1016/j.ccr.2009.11.023 PMC3711519

[27] Li R, Quan Y, Xia W. SIRT3 inhibits prostate cancer metastasis through regulation of FOXO3A by suppressing Wnt/β-catenin pathway. Experimental Cell Research. 2018;364:143-151. 10.1016/j.yexcr.2018.01.036 29421536

[28] Dds T, Kamarajan P, Joo MN, et al. Sirtuin-3 (SIRT3), a novel potential therapeutic target for oral cancer. Cancer. 2011;117:1670-1678. 21472714 10.1002/cncr.25676 PMC3117020

[29] Tong S, Fei W, Emily S, et al. SIRT3, a mitochondrial sirtuin deacetylase, Regulates mitochondrial function and thermogenesis in brown adipocytes. J Biol Chem. 2005;280:13560–13567. 15653680 10.1074/jbc.M414670200

[30] Li H, Feng Z, Wu W, et al. SIRT3 regulates cell proliferation and apoptosis related to energy metabolism in non-small cell lung cancer cells through deacetylation of NMNAT2. Int J Oncol. 2013;43:1420–1430. 24042441 10.3892/ijo.2013.2103 PMC3823398

[31] Müller W, Noguchi T, Wirtz H, et al. Expression of cell-cycle regulatory proteins cyclin D1, cyclin E, and their inhibitor p21 WAF1/CIP1 in gastric cancer. J Pathol. 2015;189:186–193. 10.1002/(SICI)1096-9896(199910)189:2<186::AID-PATH418>3.0.CO;2-L 10547573

[32] Fadaka AO, Sibuyi N, Bakare OO, et al. Expression of cyclin-dependent kinases and their clinical significance with immune infiltrates could predict prognosis in colorectal cancer. Biotechnology Reports. 2021;29:1-15. 10.1016/j.btre.2021.e00602 PMC7937668 33732631

[33] Riess C, Irmscher N, Salewski I, et al. Cyclin-dependent kinase inhibitors in head and neck cancer and glioblastoma—backbone or add-on in immune-oncology? Cancer and Metastasis review. 2021;40:153-171. 10.1007/s10555-020-09940-4 PMC7897202 33161487

[34] Nurse P. Cyclin dependent kinases and cell cycle control (Nobel Lecture). Chembiochem. 2015;3:596–603. 10.1002/1439-7633(20020703)3:7<596::AID-CBIC596>3.0.CO;2-U 12324993

[35] Grison A, Atanasoski S. Cyclins, Cyclin-dependent kinases, and cyclin-dependent kinase inhibitors in the mouse nervous system. Mol Neurobiol. 2020;57:3206–3218. 32506380 10.1007/s12035-020-01958-7

[36] Yao D, Wang P, Zhang J, et al. Deconvoluting the relationships between autophagy and metastasis for potential cancer therapy. Apoptosis. 2016;21:683–698. 27003389 10.1007/s10495-016-1237-2

[37] Radisky DC. Epithelial-mesenchymal transition. Cancer Res. 2008;68:9574. 19047131 10.1158/0008-5472.CAN-08-2316

[38] Giralt A, Villarroya F. SIRT3, a pivotal actor in mitochondrial functions: metabolism, cell death and aging. Biochem J. 2012;444:1–10. 22533670 10.1042/BJ20120030

[39] Jablonski RP, Kim SJ, Cheresh P, et al. SIRT3 deficiency promotes lung fibrosis by augmenting alveolar epithelial cell mitochondrial DNA damage and apoptosis. FASEB J. 2017;31:2520-2532. 10.1096/fj.201601077R PMC5434657 28258190

[40] Mei Y, Chunsong Y, Yuhua P. Effects of downregulation of SIRT3 expression on proliferation and apoptosis in esophageal squamous cell carcinoma EC9706 cells and its molecular mechanisms. Biomed Mater Eng. 2014;24:3883–3890. 25227106 10.3233/BME-141219

[41] Qiao A, Wang K, Yuan Y, et al. Sirt3-mediated mitophagy protects tumor cells against apoptosis under hypoxia. Oncotarget. 2016:7:43390-43400. 27270321 10.18632/oncotarget.9717 PMC5190031

[42] Zw A, Ying LB, Ying WC, et al. Pyrroloquinoline quinine protects HK-2cells against high glucose-induced oxidative stress and apoptosis through Sirt3 and PI3K/Akt/FoxO3a signaling pathway. Biochem Biophys Res Commun. 2019;508:398–404. 30502093 10.1016/j.bbrc.2018.11.140

[43] Wong WL, Puthalakath H. Bcl-2 family proteins: the sentinels of the mitochondrial apoptosis pathway. Iubmb Life. 2010;60:390–397. 10.1002/iub.51 18425793

